# Characterizing Relationships between T-cell Inflammation and Outcomes in Patients with High-Risk Neuroblastoma According to Mesenchymal and Adrenergic Signatures

**DOI:** 10.1158/2767-9764.CRC-24-0214

**Published:** 2024-08-28

**Authors:** Maria E. Kaufman, Omar R. Vayani, Kelley Moore, Alexandre Chlenski, Tong Wu, Sang Mee Lee, Ami V. Desai, Chuan He, Susan L. Cohn, Mark A. Applebaum

**Affiliations:** 1 The University of Chicago Pritzker School of Medicine, Chicago, Illinois.; 2 Department of Pediatrics, Section of Hematology/Oncology, The University of Chicago, Chicago, Illinois.; 3 Department of Chemistry, University of Chicago, Chicago, Illinois.; 4 Biostatistics Laboratory and Research Computing Group, The University of Chicago, Chicago, Illinois.; 5 Howard Hughes Medical Institute, Chevy Chase. Maryland.

## Abstract

Recent insights have identified adrenergic (ADRN) and mesenchymal (MES) cell lineages as distinct biologic cell types and T-cell inflammation as a prognostic marker in neuroblastoma. We hypothesized that elucidating unique and overlapping aspects of these biologic features could serve as novel biomarkers for informing ongoing efforts to improve therapeutic approaches for children with high-risk neuroblastoma. We identified lineage-specific, single-stranded super-enhancers to define ADRN and MES specific genes. Publicly available RNA-seq of diagnostic tumor biopsies was used in Discovery and Validation cohorts. Each tumor was assigned a relative MES score and T-cell inflammation (TCI) score. Survival was assessed using the Kaplan–Meier method, and differences were assessed by the log-rank test. Inflammation scores were correlated with MES scores and anticorrelated with *MYCN*-amplification in both cohorts. Among patients with high-risk, ADRN tumors, those with TCI tumors had superior overall survival to those with non-inflamed tumors. A similar, but nonsignificant, trend was observed in the Validation cohort. Conversely, there was no difference according to TCI status in the MES cohort in either the Discover or Validation cohorts. High-inflammation scores were correlated with improved survival in some patients with high-risk, ADRN but not MES neuroblastoma. Our findings bolster support for further developing T-cell-based and immunotherapy-based approaches for children with high-risk neuroblastoma of varying MES and ADRN expression.

**Significance:**

Adrenergic (ADRN) and mesenchymal (MES) lineages are distinct biologic cell types in neuroblastoma. We defined ADRN and MES specific genes and found that high-risk, ADRN tumors harboring elevated T-cell inflammation signatures had superior overall survival. Our findings bolster support for further developing immunotherapy-based approaches for children with high-risk neuroblastoma.

## Introduction

Neuroblastoma is the most common extracranial pediatric malignancy and is hallmarked by heterogeneous patient outcomes. While low-risk tumors often spontaneously regress or differentiate, long-term survival rates for patients with high-risk neuroblastoma remain at 50% to 60% due to high rates of refractory and relapse disease (1). Immunotherapy with anti-GD2 antibodies have been approved for use in children with high-risk neuroblastoma (2, 3) but many children continue to relapse despite these advancements. Therefore, an improved knowledge of how tumor biology impacts response to therapy and ultimately survival is necessary to improve therapeutic strategies.

High-risk neuroblastoma comprises two distinct cell lineages, mesenchymal (MES) and adrenergic (ADRN), defined by modifiable super enhancers (SEs) that allow for lineage interconversion (4). Recent studies have noted that the mesenchymal lineage is associated with an immunogenic state (5, 6). Studies in numerous cancers have shown that tumors with immunogenic microenvironments have better prognoses and are more responsive to immunotherapy (7). While neuroblastoma has been considered a “cold” cancer, recent studies have shown that the immune landscape in neuroblastoma is nuanced. *MYCN-*amplified tumors, which are composed of a higher proportion of ADRN type cells, are typically associated with “cold” or low T-cell inflammation (TCI) signatures and few tumor-associated T cells (8, 9). Conversely, patients with *MYCN*-non-amplified high-risk neuroblastoma and high TCI status have been associated with improved survival (10). Furthermore, MES cell lineage has also been associated with intratumoral TCI (5), and recent studies in cell lines have demonstrated that in response to inflammatory stimuli, MES cells release proinflammatory cytokines, leading to increased T-cell killing of tumor, while adrenergic cells do not (6). Overall, these findings suggest that the relationship between *MYCN* amplification, TCI, and mesenchymal cell lineage is complex and interwoven and increased understanding of these relationships is vital to understanding the immune component of neuroblastoma.

In this study, we aimed to optimize the epigenetic characterization of neuroblastoma tumors as predominantly MES or ADRN by utilizing a novel approach combining ChIP-seq, KAS-seq, and RNA-seq to identify the most relevant lineage-specific SEs. We then examined the relationship between TCI and cell lineage and their relationship to patient survival to generate novel biomarkers to potentially aid in therapeutic development for patients with neuroblastoma.

## Materials and Methods

### Cell culture of neuroblastoma cell lines

ADRN neuroblastoma cell lines LA1-55n (RRID: CVCL_2548), SH-SY5Y (RRID: CVCL_0019), NBL-W-N (RRID: CVCL_9900), SK-N-BE2 (RRID: CVCL_0528), and NBL-S (RRID: CVCL_2136) and MES cell lines LA1-5s (RRID: CVCL_2549), SHEP (RRID: CVCL_0524), and NBL-W-S (RRID: CVCL_9901) were grown at 5% CO_2_ in RPMI 1640 medium (Gibco, 11875-093) supplemented with 10% heat-inactivated FBS (gibco, 26140-079), L-glutamine 200 mmol/L (gibco, 25030-081), and Antibiotic-Antimycotic 100× (gibco, 15240-062). Of note LA1-5s and LA1-55n, SHEP and SH-SY5Y, and NBL-W-S and NBL-W-N are isogenic pairs with respective mesenchymal and adrenergic phenotypes. Cells were counted using a hemocytometer and grown in a T225 flask for a period of 24 hours. Cells were then harvested and pelleted in 50 mL conical tubes. They were then washed with a 1× DPBS solution (gibco, 14190-144) and resuspended in 20 mL RPMI 1640 medium (gibco, 11875-093). Resuspended cells were separated into 10 mL aliquots for chromatin extraction.

LA1-55n, SH-SY5Y, NBL-W-N, SK-N-BE2, NBL-S, LA1-5s, SHEP, and NBL-W-S were obtained from Dr. Susan Cohn (University of Chicago) in 2017. Authentication of these cell lines was performed at The Johns Hopkins University Fragment Analysis Facility (Baltimore, MD) using the AmpFlSTR Identifiler PCR Amplification Kit (Applied Biosystems). Cells were cultured in a passage lower than 25 from the original stock. All cell lines tested negative for mycoplasma contamination upon first passage using the MycoAlert Detection Assay (Lonza, LT07-318).

### Chromatin extraction and ChIP-seq

Chromatin immunoprecipitation (ChIP) was performed as previously described (11) with duplicate biologic replicates. Briefly, cultured cells were fixed with 36.5% Formaldehyde (Sigma, F87750) to a final concentration of 1%. Nuclei were isolated and the DNA was sheared to 100 to 400 bp fragments. Histone-bound chromatin was immunoprecipitated using Anti-Histone H3K27ac antibody (Abcam, Cat. # ab4729, RRID: AB_2118291) and H3K4me3 antibody (Cell Signaling Technology, Cat. # 9751, RRID: AB_2616028). Crosslinking was reversed and DNA was purified using Qiagen PCR kit (Qiagen, 28104). ChIP sequencing (ChIP-seq) libraries were made using Ovation Ultralow System V2 kit (Tecan Genomics, 0344NB-32) per the manufacturer’s instructions and sequenced on an Illumina NovaSeq 6000.

### RNA-seq library construction

RNA was isolated with triplicate biologic replicates using TRIzol reagent (Life Technologies, 15596026) according to the manufacturer’s protocol. The concentration was measured using UV spectroscopy (DeNovix). DNA was removed with the TURBO DNA-free kit (Thermo Fisher Scientific, AM2238) per the manufacturer’s instructions. Ribosomal RNA was removed with the oligo-DT kit and a directional RNA library was constructed. A total of 100 base-pair, paired-end libraries were sequenced on an Illumina NovaSeq 6000. Reads underwent quality control, trimmed with Trimmomatic v0.36 (RRID: SCR_011848; ref. 12), and aligned to hg38 using STAR RNAseq aligner v2.6.1d (RRID: SCR_004463)

### KAS-seq library construction

KAS-seq libraries were generated as previously described (13) in duplicate biologic replicates. Briefly, cells were incubated in completed culture medium containing N_3_-kethoxal. Cells were collected and genomic DNA (gDNA) was isolated from cells by PureLink genomic DNA mini kit (Thermo, K182002). gDNA was sheared and fragmented to 150 to 350 bp segments. About 5% of the fragmented DNA was saved as input, and the remaining 95% was used to enrich biotin-tagged DNA. DNA was eluted and its corresponding input control were used for library construction by using Accel-NGS Methyl-seq DNA library kit (Swift, 30024). The libraries were sequenced on Illumina Nextseq500 platform with single-end 80-bp mode.

### Processing of ChIP-seq, KAS-seq, and RNA-seq

The quality for all sequencing files was verified using FASTQC v.011.5 (RRID: SCR_014583) and cleaned using Trimmomatic v0.36 (RRID: SCR_011848; ref. 12) using default settings. Reads were then aligned to GhC38 using Bowtie2 v2.3.0 (RRID: SCR_016368; ref. 14) using default settings and deduplicated with PICARD v2.8.1 (RRID: SCR_006525). Peaks were called using MACS2 v2.1.0 (RRID: SCR_013291; ref. 15) for each sample using the arguments -keep-dup = auto -broad. For the RNA-seq, sequencing quality was verified using FASTQC v.011.5 (RRID: SCR_014583) and reads were cleaned using Trimmomatic (RRID: SCR_011848) v0.36. After alignment using the STAR RNAseq aligner v2.6.1d (RRID: SCR_004463; ref. 16), lists of gene counts were generated for each cell line using featureCounts (RRID: SCR_012919; ref. 17). Reads were loaded into R using DESeq (RRID: SCR_000154) and genes differentially expressed between MES and ADRN cell lines were identified (*P*_adj_ < 0.05 and absolute log fold change >1) using DESeq2 v1.20.0 in R v3.6.0 (RRID: SCR_015687; ref. 18). For analysis of gene expression, reads were processed using a variance stabilizing transformation with the DESeq2 (RRID:SCR_015687) package.

### Compiling SEs using ROSE and identifying lineage-specific genes

Rank ordering of SEs (ROSE; RRID: SCR_017390; ref. 19) was performed for each cell line using the H3K27ac and H3K4me3 peaks with the arguments -c -s 0 -t 0. To identify SEs, any H3K27ac peaks with signal >1000 ROSE units in the H3K4me3 data were discarded as they represented transcription start sites. Peaks around the *MYCN* amplicon were also discarded. The ROSE algorithm was re-run on the remaining H3K27ac peaks to generate a list of SEs for each cell line. These SEs were then processed through the KAS-seq pipeline (13) to determine which SEs are single-stranded. All single-stranded SEs (ssSEs) for the ADRN cell lines and the MES cell lines were then combined and overlapping sequences were merged within each phenotype to create list of ssSEs for each phenotype; each ssSE only had to be identified in a single cell line to be retained. Genes within 500 kbs of the lineage-specific ssSEs were identified and retained if they also had significantly higher expression in the corresponding cell lineage. Retained genes were included as the ADRN and MES signatures. Pathway enrichment analysis was performed for the final signatures using Gene Set Enrichment Analysis (RRID: SCR_003199; ref. 20).

### Data visualization with deepTools

Matrices with the ROSE (RRID: SCR_017390) generated SE and ssSE coordinates and were created for each sample. Heatmaps were then generated for each cell line using these matrices. Both matrix and heatmap creation were done using deepTools v3.5.1 (RRID: SCR_016366; ref. 21).

### Primary tumor RNA-seq

Publicly available RNA-seq of primary neuroblastoma tumors was used in a Discovery (GSE49711) and Validation (TARGET NBL, dbGaP: phs000467.v21.p8) cohort. Reads were downloaded from the NCBI Gene Expression Omnibus (RRID: SCR_005012) and then converted to transcripts per million (TPM).

### Tumor scoring and characterization

To assess relative expression of cell lineage–specific genes within each tumor, we used Gene Set Variation Analysis (GSVA; RRID: SCR_021058), a tool which models pathway variation across samples in an unsupervised manner (22). Each sample was assigned both a MES and ADRN score using the respective gene signature based on the ssSE-associated genes as described above. An adjusted mesenchymal score (MES_adj_) was calculated by subtracting the ADRN score from the MES score, and tumors were characterized as mesenchymal (top third according to MES_adj_) or adrenergic (bottom third according to MES_adj_). These cutoffs are similar to the percentage of these respective tumor types as defined previously (5). Each tumor was also assigned a TCI score based on a published CD8^+^ T-cell gene signature (23), which has been validated in multiple tumor types (24, 25) and shown to correlate with response to immunotherapy using similar methods. The third of tumors with the highest TCI score were characterized as TCI, and the rest as non-inflamed (NI) as described by previous studies (8).

### Statistics

Survival was assessed by the Kaplan–Meier method using the log-rank test. Differences were also determined using 3-year point estimates of overall survival (OS) as well as 95% confidence interval (CI) were assessed. Hazard ratios were obtained from univariate Cox proportional hazards models. TCI versus NI and *MYCN* status as categorical variables which were then incorporated into a multivariate model that also include a TCI*MYCN interaction term. *P* values <0.05 were considered significant.

### Data availability

For the Discovery cohort, RNA-seq expression data and associated patient data were downloaded from GSE49711 (SEQC-NB dataset). For the Validation cohort, RNA-seq raw read counts generated using HTSeq (RRID: SCR_005514) and clinical data from the TARGET-NBL project were downloaded from the GDC Data Portal (RRID: SCR_014514; now available as STAR counts.)

## Results

### ssSEs identify cell lineage–specific genes

**Figure 1:**
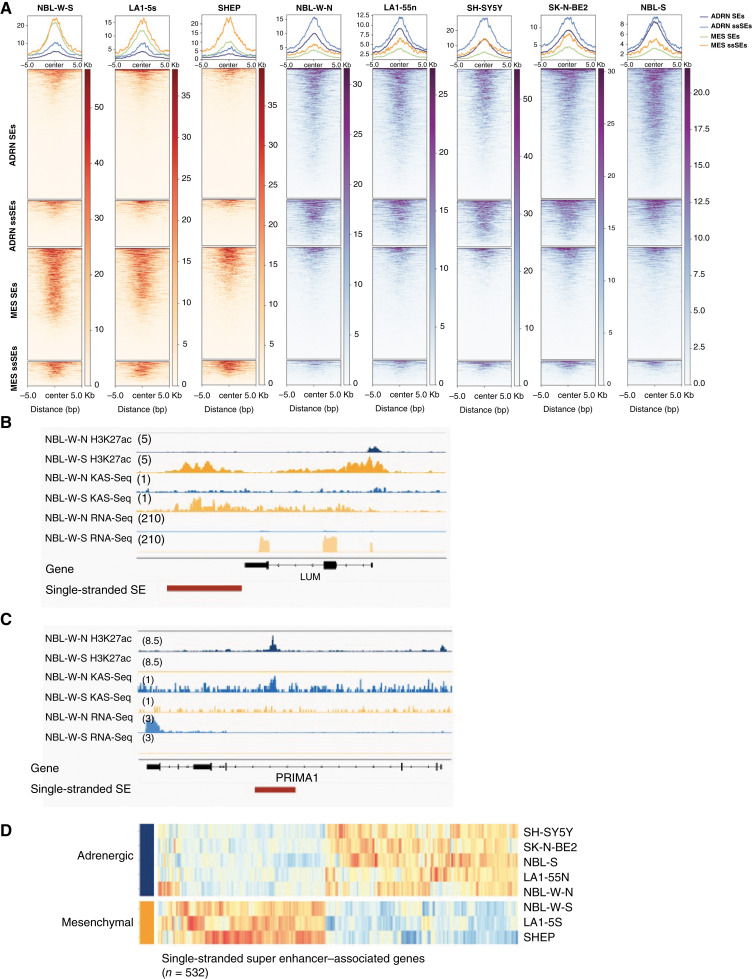
ssSEs identify highly expressed genes unique to the ADRN and MES lineages. **A,** H3K27ac deposition on ADRN and MES SEs and ssSE in each neuroblastoma cell line. Peaks are higher for ssSEs than double-stranded SEs. **B,** H3K27ac deposition, KAS-seq pulldown, and RNA expression at MES ssSE, denoted in red, associated with a MES signature gene *LUM* in representative ADRN and MES isogenic cell lines and **C,** a ADRN signature gene *PRIMA1* in the same representative cell lines. **D,** Heatmap representing 532 differentially expressed genes that were identified in five ADRN and three MES cell lines in proximity to ssSEs (*P* < 0.05).

Using ChIP-seq for H3K27ac and H3K4me3, ROSE identified 1,512 SEs in the three MES (LA1-5s, SHEP, and NBL-W-S, Supplementary Fig. S1A) and 1,951 SEs in the five ADRN (LA1-55n, SH-SY5Y, NBL-W-N, SK-N-BE2, and NBL-S, Supplementary Fig. S1B) cell lines (26, 27). Of the MES SEs, 270 overlapped with KAS-seq peaks. Of note, H3K27ac signal was higher in the MES ssSEs than double stranded SEs in the MES cell lines (Fig. 1A). There were 500 genes with increased expression in MES compared to ADRN cell lines that were within 500 kb of a SE. Of these genes, 159 were near SEs that were single-stranded and retained in the final MES signature (Fig. 1B; Supplementary Table S1). KAS-seq peaks were also identified corresponding to 512 ssSEs in the ADRN cells. Similar to what was observed with MES-specific SEs, H3K27ac signal in ADRN cells was higher in the ssSEs than double stranded SEs. There were 746 genes with increased expression in ADRN compared to MES cell lines near a SE. Of these genes, 373 were near SEs that were single-stranded and retained in the final ADRN signature (Fig. 1C and D; Supplementary Table S2). There was significant overlap of our identified gene signatures and those of the ADRN and MES signatures of van Gronigen (4) as compared to random chance including 32 MES genes (*P* = 6.4e−19) and 47 ADRN genes (*P* = 2e−23; Supplementary Table S3). Similarly, there were three overlapping MES genes (*P* = 3e−04) and three ADRN genes (*P* = 3.1e−04; Supplementary Table S3) that overlapped with those published by Boeva (28) which was significantly greater than chance relative to the size of these gene sets.

**Figure 2:**
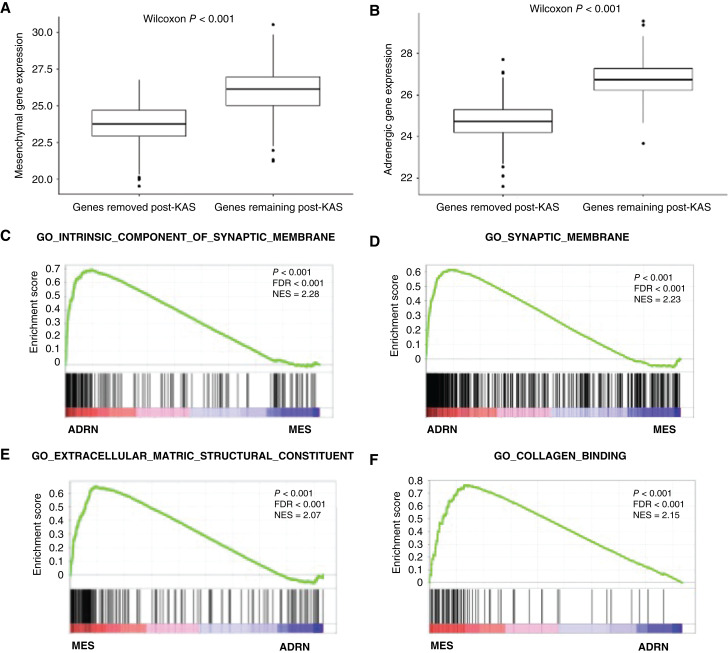
Lineage-specific genes near ssSEs have significantly higher expression and reflect tumor biology. **A,** Of the 712 genes with significantly higher expression in MES compared to ADRN cells near SEs, the 157 genes near ssSEs had higher expression in diagnostic biopsies than the 555 genes near double-stranded SEs. **B,** Of the 957 genes with significantly higher expression in MES compared to ADRN cells near SEs, the 371 genes near ssSEs had higher expression in diagnostic biopsies than the 586 genes near double-stranded SEs. **C** and **D,** ADRN signature genes were enriched for pathways related to their neural phenotype. **E** and **F,** MES signature genes were enriched for pathways related to the extracellular matrix and collagen binding as has been described for these cell types.

We obtained publicly available RNA-seq of primary neuroblastoma tumors in a Discovery (GSE49711) and Validation (TARGET NBL, dbGaP: phs000467.v21.p8) cohorts (Table 1). We confirmed that MES and ADRN signature genes had higher expression compared to differentially expressed genes near a SE that was double-stranded in 498 primary neuroblastoma tumors from the Discovery cohort. Of the 553 MES genes near a double-stranded SE, the average expression was 23.7 TPM versus 26 TPM for the 159 MES signature genes (*P* < 0.001; Fig. 2A). For the ADRN genes near a double-stranded SE, the average expression was 24.7 TPM versus 26.8 TPM in the 373 ADRN signature genes (*P* < 0.001; Fig. 2B). Similarly, in the Validation cohort of 145 patients, the MES genes near a double-stranded SE had an average expression of 50.5 TPM versus 85.9 TPM in the MES signature genes (*P* < 0.001). The ADRN genes near a double-stranded SE had an average expression of 69 TPM versus 87.8 TPM in the ADRN signature genes (*P* < 0.001). Consistent with other published signatures (4), the genes in the ADRN signature were enriched for pathways related to the synaptic membrane (Fig. 2C and D), while genes in the MES signature were enriched for pathways related to the extracellular matrix and collagen binding (Fig. 2E and F).

**Table 1:** Patient characteristics of the Discovery and Validation cohorts

Feature	Overall	High-risk subset
	*n* = 498	*n* = 176
Discovery		
Median age at diagnosis, months (IQR)	14.6 (5.4–33.2)	36.4 (23.4–54.1)
Sex		
Male	287 (57.6%)	111 (63.1%)
Female	211 (42.4%)	65 (36.9%)
Age at diagnosis		
≤ 18 months	304 (61%)	30 (17%)
18 months–5 years	149 (30%)	111 (63.1%)
> 5 years	45 (9%)	35 (19.9%)
INSS Stage		
1, 2, 3, 4s	315 (63.3%)	27 (15.3%)
4	183 (36.7%)	149 (84.7%)
MYCN-status		
Amplified	92 (18.5%)	92 (52.3%)
Non-Amplified	401 (80.5%)	83 (47.2%)
Unknown	5 (1%)	1 (0.5%)

	*n* = 145	*n* = 123
Validation		
Median age at diagnosis, months (IQR)	35.3	41.4
Sex		
Male	86 (59.3%)	69 (56.1%)
Female	59 (40.7%)	54 (43.9%)
Age at diagnosis		
≤ 18 months	23 (15.9%)	2 (1.6%)
18 months–5 years	91 (62.8%)	90 (73.2%)
> 5 years	31 (21.3%)	31 (25.2%)
INSS Stage		
1, 2, 3, 4s	25 (17.2%)	3 (2.4%)
4	120 (82.8%)	120 (97.6%)
MYCN-status		
Amplified	112 (77.2%)	90 (73.2%)
Non-Amplified	32 (22.1%)	32 (26%)
Unknown	1 (0.7%)	1 (0.8%)

### T-cell inflammation is correlated with mesenchymal cell lineage, *MYCN*-non-amplified tumor status, and lower stage in primary neuroblastoma tumors

**Figure 3:**
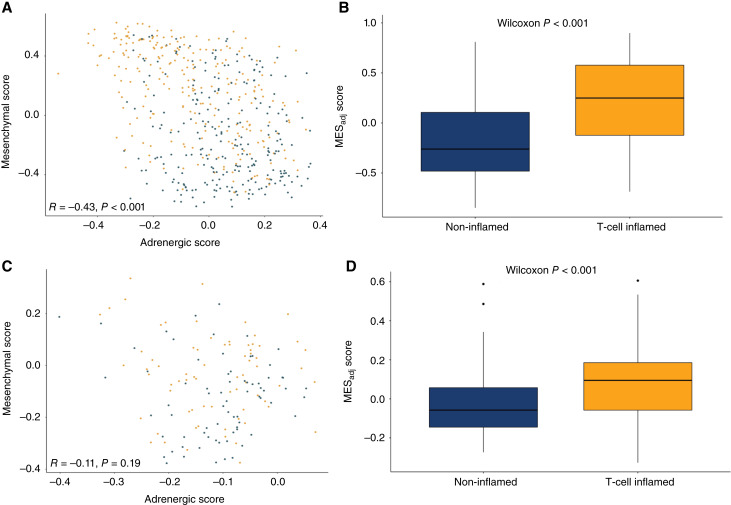
MES scores are anticorrelated with ADRN scores and correlated with TCI. **A,** Association of MES and ADRN scores in the Discovery cohort. **B,** MES_adj_ scores were also significantly higher in TCI tumors in the Discovery cohort. **C,** Similar trends for inverse correlations were seen in the Validation cohort. **D,** MES_adj_ scores were also significantly higher in TCI tumors in the Validation cohort.

Using the final signatures derived from ssSEs, each tumor was assigned a MES score and an ADRN score using GSVA. To identify tumors with the strongest MES and lowest ADRN characteristics, we assigned an adjusted mesenchymal score (MES_adj_) generated by subtracting the ADRN score from the MES score. In the Discovery cohort, MES and ADRN scores were inversely correlated (*R* = −0.43; *P* < 0.001; Fig. 3A and B). However, while the expression profiles in the Validation cohort trended similarly, the finding was not statistically significant (*R* = −0.11; *P* = 0.2). In both the Discovery and Validation cohorts, TCI tumors had significantly higher MES_adj_ scores than NI tumors (*P* < 0.001 and *P* < 0.001, respectively; Fig. 3C and D).

**Figure 4:**
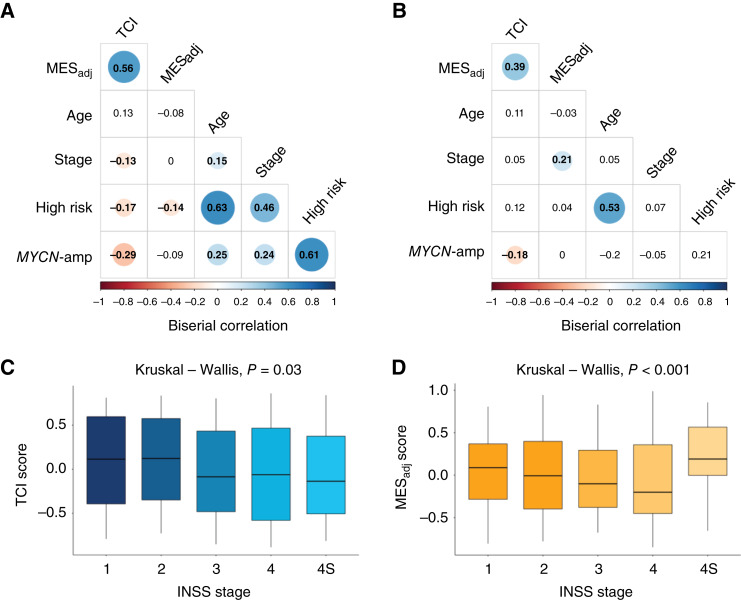
MES_adj_ and TCI scores are inversely correlated with features of high-risk neuroblastoma. MES_adj_ and TCI are correlated in both the (**A**) Discovery and (**B**) Validation cohorts and inversely correlated with *MYCN* amplification, stage, and age in the Discovery cohort. Colored circle representing the correlation coefficient shown only for those correlations with *P* < 0.05. In the Discovery cohort, TCI and MES_adj_ decrease with increasing stage (**C** and **D**), although 4S tumors have lower TCI and higher MES_adj_.

We also examined the relationship between TCI, MES_adj_, and other available clinical and biological features. In both the Discovery and Validation cohorts, TCI was positively correlated with MES_adj_ (Spearman correlation = 0.56, *P* < 0.001 and 0.38, *P* < 0.001, respectively). TCI was inversely correlated with *MYCN* amplification in both the Discovery (biserial correlation = −0.29, *P* < 0.001; Fig. 4A) and Validation (biserial correlation = −0.18, *P* = 0.03; Fig. 4B) cohorts. TCI and MES_adj_ were lower in high-risk tumors in the Discovery cohort (*P* < 0.001 and *P* = 0.002, respectively), but this result was not observed in the Validation cohort. TCI was also inversely correlated with higher International Neuroblastoma Staging System (INSS) stage (ANOVA F = 2.64, *P* = 0.03, Fig. 4C) in the Discovery cohort, although stage 4S tumors also had low scores. Similarly, MES_adj_ was highest in stage 1 tumors and decreased by stage (*P* < 0.001; Fig. 4D), with 4S tumors also having high scores. The Validation cohort lacked low stage patients and no difference in TCI or MES_adj_ score was found between stage 3 and 4 tumors.

### T-cell inflammation is associated with improved survival in high-risk patients with adrenergic tumors

A persistent clinical challenge is to further risk stratify high-risk patients according to likely benefit from standard of care therapy (29). To evaluate the potential for our MES and ADRN signatures to accomplish this, we examined the relationship between tumor characteristics and outcomes in subcohorts consisting only of high-risk patients (Table 1). Based on prevalences provided by prior studies (8), we characterized tumors in the high-risk Discovery cohort as MES (third with highest MES_adj_, *n* = 59) or ADRN (third with lowest MES_adj_, *n* = 59), and TCI (two thirds with highest TCI score, *n* = 117) or NI (third with lowest TCI score, *n* = 59; Table 2). As expected, the MES cohort was enriched for TCI (*n* = 57, 96.6%), with just two patients in the NI group. Conversely, the ADRN cohort was mostly NI (*n* = 37, 62.7%). This association was also observed in the Validation cohort, with the MES tumors (*n* = 33) being mostly TCI (*n* = 29, 87.8%). The ADRN tumors (*n* = 32, 50%) were evenly split according to inflammation status in this cohort.

**Table 2:** Inflammation and cell state characteristics of the Discovery and Validation cohorts

Feature	High-Risk subset
	*n* = 176
Discovery	
Inflammation state	
T-cell inflamed (TCI)	117
Non-inflamed (NI)	59
Cell state	
Mesenchymal	59
Adrenergic	59

	*n* = 123
Validation	
Inflammation state	
T-cell inflamed (TCI)	82
Non-inflamed (NI)	41
Cell state	
Mesenchymal	41
Adrenergic	41

**Figure 5:**
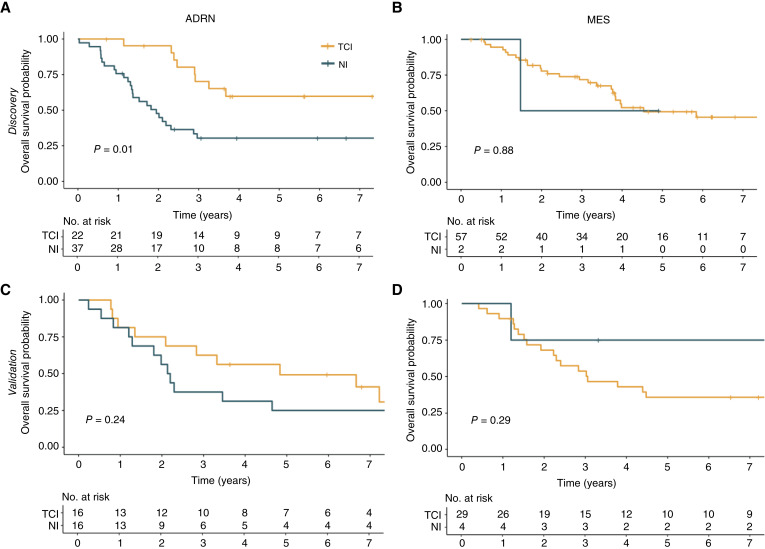
Inflammation score may be associated with for OS in high-risk, adrenergic tumors. **A,** OS was significantly better for TCI tumors than NI tumors in ADRN patients in the Discovery cohort and **B,** trended similarly in the Validation cohort. **C,** There was no difference in survival for patients with MES tumors according to TCI status in the Discovery cohort. **D,** the MES patients in the Validation also had no difference in OS according to the TCI status.

In the high-risk cohorts, there was no significant difference in survival based on MES_adj_ score alone. However, in the Discovery cohort, the 22 high-risk patients with ADRN and TCI tumors had superior OS compared to 37 patients with ADRN and NI tumors (3-year OS 70%, 95% CI 53% to 93% vs. 30%, 95% CI, 19% to 50%; *P* = 0.01; Fig. 5A). Univariate Cox regression models showed that both TCI and MYCN status were significantly associated with OS (HR, 2.57; 95% CI, 1.22–5.41 and 2.43; 95% CI, 1.23–4.82; *P* values = 0.013 and 0.011, respectively). In multivariate testing, neither inflammatory status (*P* = 0.08), MYCN (*P* = 0.11) nor an interaction between the two (*P* = 0.35) were significantly associated with OS. Thus, inflammatory status and MYCN status are associated with OS and do not appear to interact. In the Validation cohort, we found that high-risk patients with tumors having ADRN and TCI (*n* = 16) signatures trended toward better OS than those with ADRN and NI (n = 16) signatures (3-year OS 63%, 95% CI, 43%–91% vs. 38%, 95% CI, 20%–70%; *P* = 0.24), although this result was not significant likely due to small sample size (Fig. 5B). Conversely, MES tumors showed no difference in OS according to inflammatory status (*P* = 0.88; Fig. 5C). Patients with high-risk, MES tumors again had no difference in OS according to inflammation group in the Validation cohort (*P* = 0.29; Fig. 5D).

**Figure 6:**
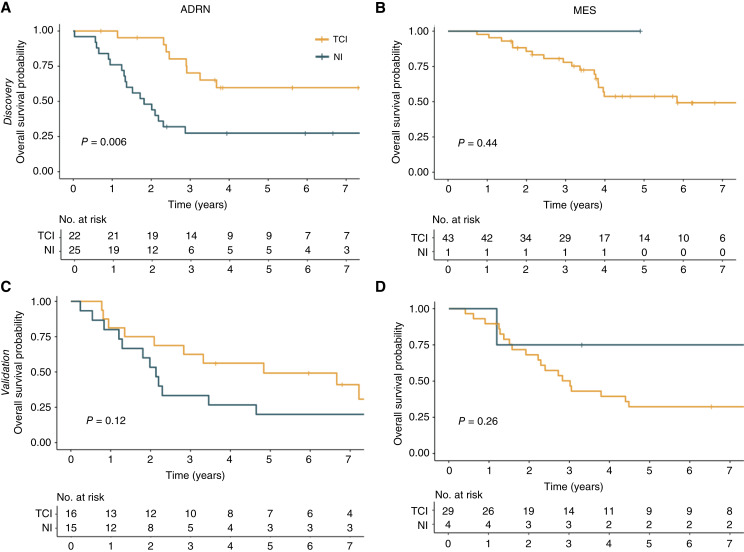
Inflammation score may be associated with OS in adrenergic tumors of patients >18 months with stage IV disease. **A,** OS was significantly better for TCI tumors than NI tumors in ADRN patients in this subgroup of the Discovery cohort and **B,** trended similarly in the Validation cohort. **C,** There was no difference in survival for patients with MES tumors according to TCI status in this subgroup in the Discovery cohort. **D,** The MES patients in this subgroup in the Validation cohort also had no difference in OS according to the TCI status.

To confirm the relevance of the patients with more uniform clinical characteristics, we performed a subgroup analysis limited to patients with high-risk disease aged >18 months at diagnosis with stage-4 disease. We similarly observed that the 22 patients with ADRN and TCI tumors had superior OS compared to 25 patients with ADRN and NI tumors (3-year OS 70.2%, 95% CI, 52.8%–93.3% vs. 27.4%; 95% CI, 14.4%–52.4%; *P* = 0.006; Fig. 6A). In the Validation cohort, we found that patients with tumors having ADRN and TCI (*n* = 16) signatures again trended towards better OS than those with ADRN and NI (*n* = 15) signatures (3-year OS 60.0%, 95% CI, 39.7%–90.7% vs. 33.3%; 95% CI, 16.3%–68.2%; *P* = 0.12; Fig. 6B). Conversely, MES tumors in this cohort showed no difference in OS according to inflammatory status in either the Discovery or Validation cohorts (*P* = 0.44 and 0.26, respectively; Fig. 6C and D).

## Discussion

We generated novel ADRN and MES lineage specific gene signatures in from neuroblastoma cell lines by incorporating transcriptionally engaged genes near sssSEs identified by KAS-seq analysis. KAS-seq is capable of rapidly assessing changes in ssDNA and could be used in future work to understand the dynamic chromatin architecture involved in cellular response to various experimental conditions evaluating mesenchymal and adrenergic phenotypes. By incorporating information of ssSEs, we found gene sets that were more highly expressed in cell lines and patient tumors. We also validate many genes incorporated by previously published signatures in an orthogonal manner using a novel technology. We further show that the MES signature is strongly correlated with a T-cell inflamed signature whereas the ADRN signature is correlated with a non-immunogenic environment, though a subset of patients had tumors that went against this trend. While we did not find a correlation of cell phenotype signatures with outcomes in patients with high-risk disease, TCI signatures were correlated with outcome for patients with high-risk, ADRN tumors in the Discovery cohort with similar, though non-statistically significant, trends in the Validation cohort, but not among patients with MES tumors. However, further studies are needed to validate this relationship given low patient numbers in our datasets and fully account for the association of MYCN with outcome.

Biologic heterogeneity is a hallmark characteristic of neuroblastoma. Importantly, neuroblastoma cells are capable of interconverting between MES and ADRN with corresponding changes in phenotype and chemosensitivity (4, 30, 31). Furthermore, recent studies demonstrated that neuroblastoma tumors enriched with MES cells are associated with relapse, and several studies have focused on converting cellular phenotype to alter therapeutic sensitivity (32–34). While others have identified lineage-specific gene signatures using lineage-specific SEs (4, 35), we also utilized KAS-seq data and incorporated genes with ssSEs, a marker of active transcription, that were differentially expressed in the phenotypically distinct neuroblastoma cell types. This identified a unique set of genes more highly expressed than those identified only by analysis of classical histone marks, suggesting these genes may play a more biologically relevant role in neuroblastoma pathogenesis.

Improving our understanding of MES and ADRN phenotypes and their role in the pathophysiology of neuroblastoma tumors has been of intense interest (36). While neuronal and stromal cellular types have been recognized for decades (37), only in the past few years has their relationship to patient tumors has come more into focus (32). Core regulatory circuitries for ADRN cells are well defined and include genes such as *ASCL1*, *DBH*, *ISL2*, and *DLK1*, all of which were identified in our initial analysis; though, *ASCL1* was eliminated during the KAS-seq pipeline (31, 38). However, beyond the key *PRRX1* gene, lineage-defining circuitries in MES cells have been harder to elucidate, suggesting a more complex network (35). Single-cell sequencing experiments have verified that these two lineages do interconvert, but certain cells may be in a more committed ADRN path (39). Furthermore, conflicting evidence exists regarding the prognostic significance of signatures identifying the abundance of these cells in patient tumors. MES signatures in tumors at diagnosis have been shown to correlate with improved outcomes (32), but relapsed tumors and therapy-resistant PDX models also have higher MES signature expression (40, 41). In this study, we confirm that defining a precise relationship between ADRN and MES signatures in bulk sequencing and outcome is challenging, likely due to the relatively low abundance of MES cells in tumors due to their more senescent nature (42).

In both Discovery and Validation cohorts, we also identified an association between MES signatures and TCI signature. Furthermore, TCI signatures were associated with improved survival amongst patients with high-risk, ADRN type tumors in the Discovery cohort though this finding was unable to be statistically validated in our smaller secondary cohort. This is consistent with a publication from Sengupta and colleagues (5) where, using the same Discovery cohort, they identified that the tumors with higher MES scores were associated with activated immune states. They further identified that MES cells promote TCI through cytokine secretion, are response to PD-L1 inhibition and are killed by cytotoxic T cells. *MYCN* amplification in tumors has been shown to be correlated with non-TCI tumors (8, 43). Specifically, Bao and colleagues identified T-cell signatures that were predictive of outcome in neuroblastoma patients in a *MYCN*-independent fashion. We further extend this work by evaluating the interaction of inflammation and cell state, as well as their relationship to other relevant tumor characteristics.

However, in neuroblastoma, it is important to note that the FDA-approved antibodies for this disease target the GD2 cell surface glycolipid. The mechanism of this antibody is thought to mediate by the activation of NK cells which was not assessed in this study. Unfortunately, genomic datasets linked to anti-GD2 treatment status are lacking, limiting our ability to evaluate how NK cells or other immune signatures are correlated to response with this modality. There is also interest in the field in CAR-T and PD-1 inhibition, which are dependent on T cells, as potential therapies for neuroblastoma. While there are also no genomic datasets linked to these therapies in patients with neuroblastoma, this study may inform which patient populations might be best suited to receive these therapies in experimental trials. However, this would need to be tested prospectively in patients treated in a uniform manner with therapies that engage T cells.

In conclusion, we developed MES and ADRN signatures based on ssSEs which identified a set of genes highly expressed in both Discovery and Validation cohorts. We confirm the association of MES signatures and TCI and show correlations with improved outcomes in a Discovery cohort for patients with tumors that have both TCI and ADRN signatures. Our findings bolster support for further developing immunotherapy-based approaches for children with high-risk neuroblastoma of varying MES and ADRN expression.

## Supplementary Material

Supplementary Figure S1 Hockey stick plots representing H3K27ac signal strength and enhancer rank to identify super-enhancers in each of the neuroblastoma cell lines.

Supplementary Table S1 159 genes identified near single-stranded super-enhancers associated with mesenchymal cell state.

Supplementary Table S2 373 genes identified near single-stranded super-enhancers associated with adrenergic cell state.

Supplementary Table S3 Table comparing our novel adrenergic and mesenchymal gene signatures with previously published signatures by Van Gronigen et al and Boeva et al.
