# Supplementary figures and images for: Characterizing Relationships between T-cell Inflammation and Outcomes in Patients with High-Risk Neuroblastoma According to Mesenchymal and Adrenergic Signatures
Source: Cancer Res Commun. 2024 Aug 28;4(8):2255–66. doi: 10.1158/2767-9764.CRC-24-0214 (PMC11350481; doi:10.1158/2767-9764.CRC-24-0214)

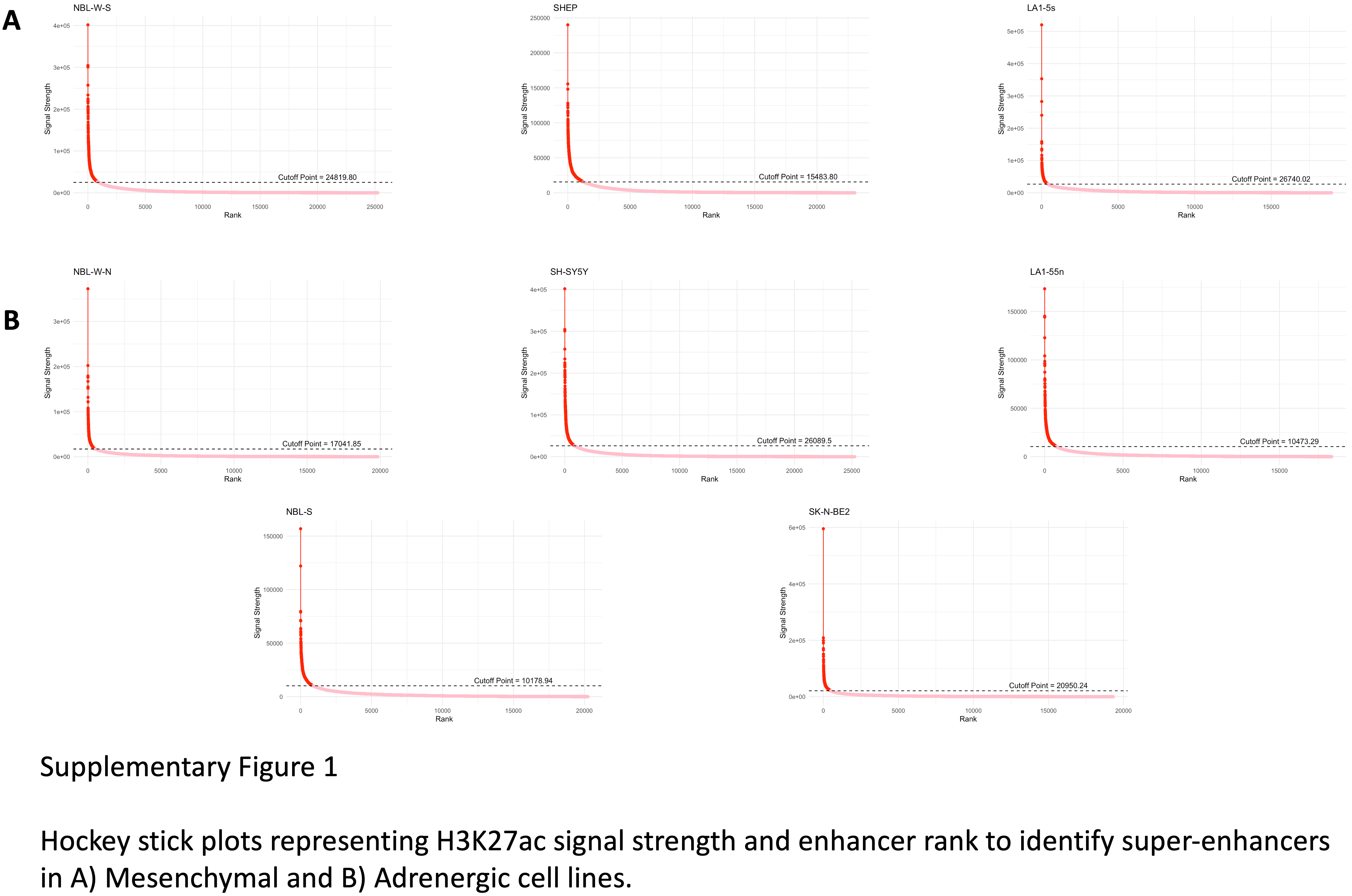

Supplement: Supplementary Figure S1 — Hockey stick plots representing H3K27ac signal strength and enhancer rank to identify super-enhancers in each of the neuroblastoma cell lines. [file crc-24-0214_supplementary_figure_s1_suppsf.png]
