# Supplementary material for: Characterizing Relationships between T-cell Inflammation and Outcomes in Patients with High-Risk Neuroblastoma According to Mesenchymal and Adrenergic Signatures
Source: Cancer Res Commun. 2024 Aug 28;4(8):2255–66. doi: 10.1158/2767-9764.CRC-24-0214 (PMC11350481; doi:10.1158/2767-9764.CRC-24-0214)
Supplement: Supplementary Table S1 — 159 genes identified near single-stranded super-enhancers associated with mesenchymal cell state. [file crc-24-0214_supplementary_table_s1_supps1.docx]

| **Supplementary Table 1.** 159 genes identified near single-stranded super-enhancers associated with mesenchymal cell state. | | | | | |
| --- | --- | --- | --- | --- | --- |
| A4GALT | CNIH3 | FKBP9 | MICALL2 | SGCA | ZCCHC24 |
| AADACL4 | COL18A1 | FOSL1 | MLPH | SH3BP2 | ZFP36L1 |
| ABCC3 | COL1A1 | FZD2 | MRC2 | SH3BP4 | ZFPM2 |
| ABHD4 | COL27A1 | GADD45B | MSLN | SIK2 | ZFYVE28 |
| ACSF2 | COL5A1 | GATA6 | MSRB3 | SIRPB1 |  |
| ACTN1 | COL6A1 | GHRL | MX1 | SIRPD |  |
| ACTN4 | CREB3L2 | GIMAP2 | MYH9 | SLC16A3 |  |
| AHRR | CRYAB | GPNMB | MYL12A | SLC9A3 |  |
| AJUBA | CSPG4 | GRB7 | MYO1C | SMAD3 |  |
| ALX4 | CTSD | GRN | MYOM1 | SMARCD3 |  |
| ANXA13 | DENND2A | HECW1 | NFIL3 | SMIM3 |  |
| ANXA8L1 | DHRS3 | HIC1 | NFKB2 | SPDL1 |  |
| APOA1 | DLX4 | HSPG2 | NID1 | STING1 |  |
| APOL2 | DNASE2 | IFI35 | NUAK2 | SYNJ2 |  |
| APOL3 | DNMBP | IFITM1 | P4HA2 | TAGLN |  |
| ARHGEF17 | DOCK2 | IFITM3 | P4HB | TFAP2C |  |
| ATP2B4 | DUSP1 | IGFBP6 | PDE6G | TGM2 |  |
| BMP1 | EFEMP2 | IRAK2 | PDGFA | TLCD2 |  |
| C11orf21 | EFHC1 | IRF1 | PGF | TMEM184A |  |
| C11orf68 | EGFR | JPH2 | PGGHG | TMEM92 |  |
| C11orf88 | EPHA2 | KANK2 | PIEZO1 | TNFRSF9 |  |
| C1QTNF1 | EPS8L2 | KIRREL1 | PITX3 | TNS2 |  |
| CALR | ERICH5 | KLF2 | PLS3 | TNS3 |  |
| CCNO | ERRFI1 | KLHL38 | PODXL | TOM1L2 |  |
| CD151 | EVA1B | KRT18 | POLR2L | TOR4A |  |
| CD59 | EXT1 | KRT8 | RAD51B | TRAM2 |  |
| CD81 | EXT2 | LDLRAD2 | RIN2 | TSPAN10 |  |
| CDC20B | FANK1 | LGALS3BP | RNF213 | TSPAN32 |  |
| CFI | FBF1 | LTBP2 | RUNX1 | TSPAN4 |  |
| CKAP4 | FBLIM1 | LUM | SAMD15 | UPK3B |  |
| CLEC4M | FBXO32 | MAFK | SEMA7A | VMP1 |  |
