# Supplementary material for: Characterizing Relationships between T-cell Inflammation and Outcomes in Patients with High-Risk Neuroblastoma According to Mesenchymal and Adrenergic Signatures
Source: Cancer Res Commun. 2024 Aug 28;4(8):2255–66. doi: 10.1158/2767-9764.CRC-24-0214 (PMC11350481; doi:10.1158/2767-9764.CRC-24-0214)
Supplement: Supplementary Table S2 — 373 genes identified near single-stranded super-enhancers associated with adrenergic cell state. [file crc-24-0214_supplementary_table_s2_supps2.docx]

| **Supplementary Table 2.** 373 genes identified near single-stranded super-enhancers associated with adrenergic cell state. | | | | | | | | |
| --- | --- | --- | --- | --- | --- | --- | --- | --- |
| ABCD2 | CELF5 | DTX1 | GOLT1A | KHK | NRXN2 | RALGPS1 | SHF | TIAM1 |
| ACE | CFAP20 | DUOX1 | GPR153 | KIAA1614 | NT5C1A | RAP1GAP | SIL1 | TIGD3 |
| ACP4 | CFAP65 | DUOXA1 | GPR61 | KIF1A | NTNG1 | RAP1GAP2 | SKI | TIPRL |
| ACTB | CHD5 | EIPR1 | GPRIN1 | KIF3A | NTRK1 | RASAL1 | SLC22A7 | TLE6 |
| ACVR1B | CHIT1 | EMC1 | GRHPR | KLF1 | PAK5 | RASSF10 | SLC25A29 | TLN1 |
| ADAMTSL2 | CHMP6 | EML4 | GRIK3 | KLF7 | PAQR6 | RBP7 | SLC29A3 | TMED10 |
| ADORA1 | CHRNA2 | EML5 | GRK3 | KPTN | PAXIP1 | RECK | SLC30A3 | TMEM151B |
| ADRA2A | CHRNA3 | ENHO | GTF3C2 | LMAN2 | PCDH1 | REEP5 | SLC35D3 | TMEM198 |
| ALK | CHRNB4 | EPHA8 | GTPBP3 | LMO1 | PCDHA1 | RELL2 | SLC39A1 | TMEM39A |
| ALLC | CHST13 | ESRRG | H1-10 | LMO2 | PCDHB11 | RGS5 | SLC4A1AP | TMEM63B |
| ANK1 | CHST8 | EXOSC6 | HAND1 | LNPK | PCDHB12 | RHBG | SLC8A2 | TMEM63C |
| ANKLE1 | CIB4 | FABP6 | HAND2 | LOC150051 | PCDHB6 | RHEBL1 | SLIT1 | TPO |
| ANKRD44 | CLASP1 | FAM124A | HECTD4 | LOXHD1 | PDE2A | RHOA | SNAP25 | TRAPPC12 |
| AQP10 | CLPB | FAM163A | HHIPL1 | LRRC71 | PDE4B | RIC3 | SNCB | TRAPPC3 |
| ARF3 | CLSTN1 | FAM163B | HIP1 | LSM4 | PDE4C | RIMBP2 | SOBP | TRMT61B |
| ARID3B | CMPK2 | FAM49A | HK3 | MAP6 | PDE6D | RITA1 | SPATS2 | TSACC |
| ASPDH | CNGB1 | FANCC | HOTS | MAP7 | PDXP | RNASEH1 | SPTBN4 | TSPAN18 |
| ATCAY | CNNM2 | FBLL1 | HOXD1 | MAST1 | PEAR1 | RNF150 | SRRM3 | TSTD2 |
| ATP1B1 | CNTN2 | FBP1 | HOXD3 | MCU | PEX7 | RNF165 | SSTR2 | TTBK1 |
| ATP5MD | COX17 | FBXL15 | HOXD4 | MFAP3 | PGAP1 | RNF216 | ST3GAL3 | TTC26 |
| ATPSCKMT | COX4I2 | FBXO10 | HPCA | MFSD13A | PHF13 | RPH3A | ST6GALNAC4 | TTC9B |
| B3GALT5 | COX7A2L | FBXO8 | HPCAL4 | MGST2 | PHOSPHO2-KLHL23 | RPTOR | ST8SIA2 | TUBA1A |
| BCL10 | CRABP1 | FEM1A | IGFBPL1 | MSH2 | PHYHIP | RRM2 | STK40 | UBA52 |
| BEGAIN | CRIP3 | FEV | IGSF11 | MTA3 | PIK3AP1 | RSAD2 | STMN4 | UHMK1 |
| BMP7 | CRYBA2 | FGD3 | IL20RA | MTSS2 | PLA2G1B | RTBDN | STXBP1 | UNC13A |
| BRD3OS | CSNK1D | FGF17 | ILK | MXI1 | PLCH2 | RTL1 | SULF2 | UNC5A |
| BSN | CTTN | FGF19 | INSM2 | MYCBPAP | PLD5 | RUBCN | SURF4 | UNC79 |
| BTBD17 | DBH | FLCN | INTS3 | MYT1L | PLPPR3 | RUNDC3A | SVBP | UQCRHL |
| C10orf95 | DDC | FLII | IPO13 | N4BP2 | PPM1G | SAMD1 | SVOP | VAV3 |
| CACNA2D2 | DDX25 | FNIP1 | IQCC | NACC1 | PPT1 | SCARA5 | SWI5 | VSTM2L |
| CACNG1 | DEGS2 | FOXN3 | ISL1 | NADK | PRIMA1 | SCML4 | SYT11 | WASF2 |
| CACNG5 | DIO3 | FOXN4 | ISLR2 | NAPSA | PRKCZ | SCN3B | SYT13 | WSCD2 |
| CALM2 | DLK1 | GAL | JAKMIP3 | NEURL1 | PROX1 | SCN8A | SYT14 | XKR7 |
| CBLN1 | DMTN | GAP43 | JPH3 | NFASC | PRR29 | SCP2 | SYT3 | XRCC5 |
| CCDC121 | DNAJB8 | GATA2 | KATNB1 | NGB | PRR36 | SCYL1 | SYT7 | ZDHHC22 |
| CCDC188 | DNAJC5G | GBGT1 | KCNC3 | NIFK | PRRT4 | SEC14L5 | SZT2 | ZFHX3 |
| CCDC92B | DOK4 | GFRA3 | KCNH4 | NKIRAS2 | PSD | SEZ6L | TBX4 | ZNF536 |
| CCM2L | DPH2 | GGA3 | KCNH6 | NMNAT2 | PTCH1 | SGIP1 | TFCP2L1 | ZNF557 |
| CCNA1 | DPT | GLRA3 | KCNK12 | NOL10 | PTPRN | SGSM1 | TH | ZNF562 |
| CDHR2 | DPYSL2 | GNB1 | KCNK3 | NPLOC4 | PURA | SHANK1 | THADA | ZNF74 |
| CDK5R2 | DRD2 | GNG4 | KCNK9 | NPTN | RAB3A | SHANK2 | THEG | ZNF780B |
