# Supplementary material for: Characterizing Relationships between T-cell Inflammation and Outcomes in Patients with High-Risk Neuroblastoma According to Mesenchymal and Adrenergic Signatures
Source: Cancer Res Commun. 2024 Aug 28;4(8):2255–66. doi: 10.1158/2767-9764.CRC-24-0214 (PMC11350481; doi:10.1158/2767-9764.CRC-24-0214)
Supplement: Supplementary Table S3 — Table comparing our novel adrenergic and mesenchymal gene signatures with previously published signatures by Van Gronigen et al and Boeva et al. [file crc-24-0214_supplementary_table_s3_supps3.docx]

| **Supplementary Table 3.** Table comparing our novel adrenergic and mesenchymal gene signatures with previously published signatures. | | | | | | | | |
| --- | --- | --- | --- | --- | --- | --- | --- | --- |
| **Unique Post-KAS ADRN genes** | | | **ADRN Overlap with van Gronigen** | **Unique Van Gronigen ADRN genes** | | | **ADRN Overlap with Boeva** | **Unique Boeva ADRN genes** |
| **n=325** | | | **n=47** | **n=322** | | | **n=3** | **n=4** |
| ARID3B | HOXD1 | RTBDN | ACVR1B | ABCA3 | GGH | PRIM1 | HAND2 | PHOX2B |
| ABCD2 | HOXD3 | RTL1 | ALK | ABCB1 | GLCCI1 | PRPH | ISL1 | GATA3 |
| ACE | HOXD4 | RUBCN | ATCAY | ABLIM1 | GLDC | PRSS12 | KLF7 | HAND1 |
| ACP4 | HPCA | SAMD1 | BMP7 | ACOT7 | GLRX | PRSS3 |  | PHOX2A |
| ACTB | HPCAL4 | SCARA5 | CACNA2D2 | ACTL6B | GMNN | PTS |  |  |
| ADAMTSL2 | IGSF11 | SCML4 | CHRNA3 | ADAM22 | GPR22 | QDPR |  |  |
| ADORA1 | IL20RA | SCN3B | DBH | ADCYAP1R1 | GPR27 | RAB33A |  |  |
| ADRA2A | ILK | SCN8A | DDC | ADGRB3 | GRB10 | RAB6B |  |  |
| ALLC | INTS3 | SCP2 | DLK1 | ADRBK2 | GRIA2 | RALGDS |  |  |
| ANK1 | IPO13 | SCYL1 | DPYSL2 | AGTPBP1 | H1FX | RANBP1 |  |  |
| ANKLE1 | IQCC | SEC14L5 | EML4 | AHSA1 | HAND2-AS1 | RBBP8 |  |  |
| ANKRD44 | ISLR2 | SEZ6L | ESRRG | AKAP1 | HES6 | RBMS3 |  |  |
| AQP10 | JAKMIP3 | SGIP1 | FABP6 | AKAP12 | HEY1 | RBP1 |  |  |
| ARF3 | JPH3 | SGSM1 | FAM163A | ANK2 | HK2 | REC8 |  |  |
| ASPDH | KATNB1 | SHANK1 | FBLL1 | ANKRD46 | HMGA1 | REEP1 |  |  |
| ATP1B1 | KCNC3 | SHANK2 | FBXO8 | ANP32A | HMP19 | RET |  |  |
| ATP5MD | KCNH4 | SHF | FEV | AP1S2 | HN1 | RFC4 |  |  |
| ATPSCKMT | KCNH6 | SIL1 | GAL | ARHGEF7 | HNRNPA0 | RGS17 |  |  |
| B3GALT5 | KCNK12 | SKI | GAP43 | ARL6IP1 | HS6ST2 | RIMS3 |  |  |
| BCL10 | KCNK3 | SLC22A7 | GATA2 | ASCL1 | ICA1 | RNF144A |  |  |
| BEGAIN | KCNK9 | SLC25A29 | GNB1 | ASRGL1 | IGSF3 | RNF157 |  |  |
| BRD3OS | KHK | SLC29A3 | GNG4 | ATL1 | INA | RNFT2 |  |  |
| BSN | KIAA1614 | SLC30A3 | HAND1 | ATP6V0E2 | INO80C | RPS6KA2 |  |  |
| BTBD17 | KIF3A | SLC35D3 | IGFBPL1 | ATP6V1B2 | INSM1 | RTN1 |  |  |
| C10orf95 | KLF1 | SLC39A1 | INSM2 | AUTS2 | IRS2 | RTN2 |  |  |
| CACNG1 | KPTN | SLC4A1AP | ISL1 | BEND4 | KDM1A | RUFY3 |  |  |
| CACNG5 | LMAN2 | SLC8A2 | KIF1A | BEX1 | KIAA1211 | RUNDC3B |  |  |
| CALM2 | LMO1 | SNCB | KLF7 | BEX2 | KIDINS220 | SATB1 |  |  |
| CBLN1 | LMO2 | SOBP | LSM4 | BIRC5 | KIF15 | SBK1 |  |  |
| CCDC121 | LNPK | SPATS2 | MAP6 | BMPR1B | KIF21A | SCAMP5 |  |  |
| CCDC188 | LOC150051 | SPTBN4 | MXI1 | C11orf95 | KIF2A | SCG2 |  |  |
| CCDC92B | LOXHD1 | SRRM3 | NMNAT2 | C14orf132 | KIF5C | SCG3 |  |  |
| CCM2L | LRRC71 | SSTR2 | RGS5 | C3orf14 | KLC1 | SCN3A |  |  |
| CCNA1 | MAP7 | ST3GAL3 | RIMBP2 | C4orf48 | KLF13 | SEC11C |  |  |
| CDHR2 | MAST1 | ST6GALNAC4 | RNF150 | C7orf55 | KLHL13 | 45538 |  |  |
| CDK5R2 | MCU | ST8SIA2 | RNF165 | CACNA1B | KLHL23 | 45541 |  |  |
| CELF5 | MFAP3 | STK40 | RRM2 | CADM1 | KNSTRN | SERP2 |  |  |
| CFAP20 | MFSD13A | SULF2 | RUNDC3A | CAMSAP1 | L1CAM | SETD7 |  |  |
| CFAP65 | MGST2 | SURF4 | SLIT1 | CCDC167 | LEPROTL1 | SHC3 |  |  |
| CHD5 | MSH2 | SVBP | SNAP25 | CCND1 | LIN28B | SHD |  |  |
| CHIT1 | MTA3 | SVOP | STMN4 | CCNI | LINC00888 | SIX3 |  |  |
| CHMP6 | MTSS2 | SWI5 | STXBP1 | CCP110 | LMO3 | SLC10A4 |  |  |
| CHRNA2 | MYCBPAP | SYT11 | TH | CCSAP | LOC100507194 | SLC35G2 |  |  |
| CHRNB4 | MYT1L | SYT13 | TIAM1 | CD200 | LOC101928409 | SLIT3 |  |  |
| CHST13 | N4BP2 | SYT14 | UNC79 | CDC42EP3 | LRRTM2 | SNAP91 |  |  |
| CHST8 | NACC1 | SYT3 | ZNF512 | CDCA5 | LSM3 | SOX11 |  |  |
| CIB4 | NADK | SYT7 | ZNF536 | CDKN2C | LYN | ST3GAL6 |  |  |
| CLASP1 | NAPSA | SZT2 |  | CDKN3 | MAGI3 | STMN2 |  |  |
| CLPB | NEURL1 | TBX4 |  | CELF2 | MANEAL | STRA6 |  |  |
| CLSTN1 | NFASC | TFCP2L1 |  | CENPU | MAP1B | SV2C |  |  |
| CMPK2 | NGB | THADA |  | CENPV | MAP2 | SYNPO2 |  |  |
| CNGB1 | NIFK | THEG |  | CEP44 | MAPK8 | SYT1 |  |  |
| CNNM2 | NKIRAS2 | TIGD3 |  | CERK | MAPT | SYT4 |  |  |
| CNTN2 | NOL10 | TIPRL |  | CETN3 | 45362 | TACC2 |  |  |
| COX17 | NPLOC4 | TLE6 |  | CHGA | MCM2 | TAGLN3 |  |  |
| COX4I2 | NPTN | TLN1 |  | CHGB | MCM6 | TBC1D30 |  |  |
| COX7A2L | NRXN2 | TMED10 |  | CHML | MCM7 | TBPL1 |  |  |
| CRABP1 | NT5C1A | TMEM151B |  | CKB | MIAT | TCEAL7 |  |  |
| CRIP3 | NTNG1 | TMEM198 |  | CLASP2 | MMD | TDG |  |  |
| CRYBA2 | NTRK1 | TMEM39A |  | CLGN | MRPL48 | TENM4 |  |  |
| CSNK1D | PAK5 | TMEM63B |  | CRH | MSH6 | TFAP2B |  |  |
| CTTN | PAQR6 | TMEM63C |  | CRMP1 | MSI2 | THSD7A |  |  |
| DDX25 | PAXIP1 | TPO |  | CSE1L | MTCL1 | TMEM108 |  |  |
| DEGS2 | PCDH1 | TRAPPC12 |  | CXADR | MYBL2 | TMEM178B |  |  |
| DIO3 | PCDHA1 | TRAPPC3 |  | CXCR4 | MYEF2 | TMEM97 |  |  |
| DMTN | PCDHB11 | TRMT61B |  | CYFIP2 | MYO5A | TMOD1 |  |  |
| DNAJB8 | PCDHB12 | TSACC |  | CYGB | MYRIP | TMOD2 |  |  |
| DNAJC5G | PCDHB6 | TSPAN18 |  | DACH1 | NANOS1 | TMTC4 |  |  |
| DOK4 | PDE2A | TSTD2 |  | DAPK1 | NAP1L5 | TOX2 |  |  |
| DPH2 | PDE4B | TTBK1 |  | DCX | NAPB | TRAP1 |  |  |
| DPT | PDE4C | TTC26 |  | DDX39A | NARS2 | TSPAN13 |  |  |
| DRD2 | PDE6D | TTC9B |  | DIABLO | NBEA | TSPAN7 |  |  |
| DTX1 | PDXP | TUBA1A |  | DKK1 | NCAM1 | TTC8 |  |  |
| DUOX1 | PEAR1 | UBA52 |  | DNAJB1 | NCAN | TUB |  |  |
| DUOXA1 | PEX7 | UHMK1 |  | DNAJC6 | NCOA7 | TUBB2A |  |  |
| EIPR1 | PGAP1 | UNC13A |  | DNAJC9 | NCS1 | TUBB2B |  |  |
| EMC1 | PHF13 | UNC5A |  | DNER | NEFL | TUBB3 |  |  |
| EML5 | PHOSPHO2-KLHL23 | UQCRHL |  | DPYSL3 | NEFM | TUBB4B |  |  |
| ENHO | PHYHIP | VAV3 |  | DPYSL5 | NELFCD | UBE2C |  |  |
| EPHA8 | PIK3AP1 | VSTM2L |  | DTD1 | NELL2 | UBE2T |  |  |
| EXOSC6 | PLA2G1B | WASF2 |  | DUSP4 | NET1 | UCP2 |  |  |
| FAM124A | PLCH2 | WSCD2 |  | EEF1A2 | NFIL3 | VRK1 |  |  |
| FAM163B | PLD5 | XKR7 |  | EIF1B | NGRN | ZNF195 |  |  |
| FAM49A | PLPPR3 | XRCC5 |  | ELAVL2 | NNAT | ZNF22 |  |  |
| FANCC | PPM1G | ZDHHC22 |  | ELAVL3 | NOL4 | ZNF24 |  |  |
| FBP1 | PPT1 | ZFHX3 |  | ELAVL4 | NPTX2 | ZNF704 |  |  |
| FBXL15 | PRIMA1 | ZFR2 |  | EML6 | NPY | ZNF711 |  |  |
| FBXO10 | PRKCZ | ZNF385C |  | ENDOG | NRCAM | ZNF738 |  |  |
| FEM1A | PROX1 | ZNF484 |  | ENO2 | NRSN1 | ZNF91 |  |  |
| FGD3 | PRR29 | ZNF557 |  | EPB41L4A-AS1 | NSG1 | ZWILCH |  |  |
| FGF17 | PRR36 | ZNF562 |  | EVL | NUDT11 |  |  |  |
| FGF19 | PRRT4 | ZNF74 |  | EXOC5 | NUF2 |  |  |  |
| FLCN | PSD | ZNF780B |  | EYA1 | NUSAP1 |  |  |  |
| FLII | PTCH1 |  |  | FAM107B | OLA1 |  |  |  |
| FNIP1 | PTPRN |  |  | FAM155A | OLFM1 |  |  |  |
| FOXN3 | PURA |  |  | FAM167A | PARP6 |  |  |  |
| FOXN4 | RAB3A |  |  | FAM169A | PBK |  |  |  |
| GBGT1 | RALGPS1 |  |  | FAM171B | PBX3 |  |  |  |
| GFRA3 | RAP1GAP |  |  | FAM60A | PDK1 |  |  |  |
| GGA3 | RAP1GAP2 |  |  | FAXC | PEG3 |  |  |  |
| GLRA3 | RASAL1 |  |  | FHOD3 | PHF21B |  |  |  |
| GOLT1A | RASSF10 |  |  | FIGNL1 | PHOX2A |  |  |  |
| GPR153 | RBP7 |  |  | FKBP1B | PHOX2B |  |  |  |
| GPR61 | RECK |  |  | FKBP4 | PHPT1 |  |  |  |
| GPRIN1 | REEP5 |  |  | FOXM1 | PHYHIPL |  |  |  |
| GRHPR | RELL2 |  |  | FOXO3 | PIK3R1 |  |  |  |
| GRIK3 | RHBG |  |  | FSD1 | PKIA |  |  |  |
| GRK3 | RHEBL1 |  |  | FZD3 | PLPPR5 |  |  |  |
| GTF3C2 | RHOA |  |  | GABRB3 | PNMA2 |  |  |  |
| GTPBP3 | RIC3 |  |  | GATA3 | POLB |  |  |  |
| H1-10 | RITA1 |  |  | GCH1 | POPDC3 |  |  |  |
| HECTD4 | RNASEH1 |  |  | GDAP1 | PPM1E |  |  |  |
| HHIPL1 | RNF216 |  |  | GDAP1L1 | PPP1R9A |  |  |  |
| HIP1 | RPH3A |  |  | GDI1 | PPP2R3C |  |  |  |
| HK3 | RPTOR |  |  | GDPD1 | PRC1 |  |  |  |
| HOTS | RSAD2 |  |  | GGCT | PRCD |  |  |  |
| **Unique Post-KAS MES genes** | | **MES Overlap with Van Gronigen** | **Unique Van Gronigen MES genes** | | | | **MES Overlap with Boeva** | **Unique Boeva MES genes** |
| **n=124** | | **n=32** | **n=453** | | | | **n=3** | **n=12** |
| A4GALT | PGGHG | ACTN1 | A2M | ELK3 | LIPA | RGL1 | FOSL1 | BHLHE41 |
| AADACL4 | PIEZO1 | AJUBA | ABRACL | ELK4 | LITAF | RGS10 | IRF1 | FLI1 |
| ABCC3 | PITX3 | ATP2B4 | ACADVL | EMILIN1 | LIX1L | RGS3 | RUNX1 | FOSL2 |
| ABHD4 | PODXL | CD59 | ACAP2 | EMP1 | LMAN1 | RHOC |  | GLIS3 |
| ACSF2 | RAD51B | CFI | ACTA2 | ENAH | LMNA | RHOJ |  | IRF2 |
| ACTN4 | RNF213 | CKAP4 | ADAM19 | EPHA3 | LOXL2 | RIT1 |  | IRF3 |
| AHRR | SAMD15 | COL1A1 | ADAM9 | EPS8 | LPP | RNFT1 |  | MAFF |
| ALX4 | SEMA7A | COL27A1 | ADAMTS5 | ERBIN | LRP10 | RNH1 |  | MEF2D |
| ANXA13 | SGCA | COL5A1 | ADGRE5 | ERLIN1 | LRRC17 | ROBO1 |  | NR3C1 |
| ANXA8L1 | SH3BP2 | COL6A1 | ADGRG6 | ETS1 | LRRC8C | ROR1 |  | PRRX1 |
| APOA1 | SH3BP4 | CREB3L2 | AEBP1 | EVA1A | LTBP1 | RRBP1 |  | RUNX2 |
| APOL2 | SIK2 | EGFR | ALDH1A3 | EXTL2 | LUZP1 | S1PR3 |  | TBX18 |
| APOL3 | SIRPB1 | ERRFI1 | AMMECR1 | F2R | MAGT1 | SASH1 |  |  |
| ARHGEF17 | SIRPD | EXT1 | ANTXR1 | F2RL2 | MAML2 | SCPEP1 |  |  |
| BMP1 | SLC16A3 | FZD2 | ANXA1 | FAM102B | MAN2A1 | SCRG1 |  |  |
| C11orf21 | SLC9A3 | GRN | ANXA2 | FAM114A1 | MANF | SDC2 |  |  |
| C11orf68 | SMARCD3 | IGFBP6 | ANXA5 | FAM120A | MBD2 | SDC4 |  |  |
| C11orf88 | SMIM3 | KANK2 | ANXA6 | FAM129A | MBNL1 | SDCBP |  |  |
| C1QTNF1 | SPDL1 | MRC2 | APOE | FAM3C | MBTPS1 | SDF4 |  |  |
| CALR | STING1 | RIN2 | APP | FAM43A | MEOX1 | SEC14L1 |  |  |
| CCNO | TAGLN | SMAD3 | ARHGAP1 | FAM46A | MEOX2 | SEL1L3 |  |  |
| CD151 | TFAP2C | SYNJ2 | ARHGEF40 | FAT1 | MEST | SEMA3C |  |  |
| CD81 | TGM2 | TRAM2 | ARL1 | FBN1 | MGAT2 | SEMA3F |  |  |
| CDC20B | TLCD2 | TSPAN4 | ARL4A | FBN2 | MGP | 45545 |  |  |
| CLEC4M | TMEM184A | ZCCHC24 | ARMCX2 | FGFR1 | MGST1 | SERPINE2 |  |  |
| CNIH3 | TMEM92 | ZFP36L1 | ARPC1B | FIBIN | MICAL2 | SERPINH1 |  |  |
| COL18A1 | TNFRSF9 | EFEMP2 | ASPH | FILIP1L | MMP2 | SFT2D1 |  |  |
| CRYAB | TNS2 | IFITM3 | ATP10D | FKBP14 | MOB1A | SFT2D2 |  |  |
| CSPG4 | TNS3 | MYL12A | ATP1B1 | FLNA | MXRA5 | SGK1 |  |  |
| CTSD | TOM1L2 | NID1 | ATP2B1 | FLRT2 | MYADM | SH3BGRL |  |  |
| DENND2A | TOR4A | PLS3 | ATP6V0E1 | FMOD | MYDGF | SHC1 |  |  |
| DHRS3 | TSPAN10 | POLR2L | ATP8B2 | FN1 | MYL12B | SHROOM3 |  |  |
| DLX4 | TSPAN32 |  | ATXN1 | FNDC3B | MYLIP | SIX1 |  |  |
| DNASE2 | UPK3B |  | B2M | FSTL1 | NANS | SIX4 |  |  |
| DNMBP | VMP1 |  | BAG3 | FUCA2 | NBR1 | SKIL |  |  |
| DOCK2 | ZFPM2 |  | BGN | FZD1 | NEK7 | SLC16A4 |  |  |
| DUSP1 | ZFYVE28 |  | BMP5 | FZD7 | NES | SLC30A1 |  |  |
| EFHC1 |  |  | BNC2 | GABRR1 | NFIA | SLC30A7 |  |  |
| EPHA2 |  |  | BOC | GALNT10 | NFIC | SLC35F5 |  |  |
| EPS8L2 |  |  | BTN3A2 | GAS1 | NID2 | SLC38A2 |  |  |
| ERICH5 |  |  | C1orf198 | GAS2 | NOTCH2 | SLC38A6 |  |  |
| EVA1B |  |  | C1orf54 | GDF15 | NOTCH2NL | SLC39A14 |  |  |
| EXT2 |  |  | C4orf32 | GJA1 | NPC2 | SNAI2 |  |  |
| FANK1 |  |  | C6orf120 | GNAI1 | NPTN | SNAP23 |  |  |
| FBF1 |  |  | CALD1 | GNG12 | NQO1 | SOSTDC1 |  |  |
| FBLIM1 |  |  | CALU | GNS | NR3C1 | SOX9 |  |  |
| FBXO32 |  |  | CAPN2 | GORAB | NRP1 | SPARC |  |  |
| FKBP9 |  |  | CAPN6 | GPC6 | OGFRL1 | SPARCL1 |  |  |
| GADD45B |  |  | CBFB | GPR137B | OLFML2A | SPATA20 |  |  |
| GATA6 |  |  | CBLB | GPX8 | OLFML2B | SPCS3 |  |  |
| GHRL |  |  | CCDC80 | GSN | OLFML3 | SPRED1 |  |  |
| GIMAP2 |  |  | CD164 | HES1 | OSTC | SPRY1 |  |  |
| GPNMB |  |  | CD44 | HEXB | P4HA1 | SPRY4 |  |  |
| GRB7 |  |  | CD63 | HIBADH | PALLD | SPRY4-IT1 |  |  |
| HECW1 |  |  | CDH11 | HIPK3 | PAPSS2 | SQSTM1 |  |  |
| HIC1 |  |  | CETN2 | HIST1H2AC | PCDH18 | SRPX |  |  |
| HSPG2 |  |  | CFH | HIST1H2BK | PCOLCE2 | SSBP4 |  |  |
| IFI35 |  |  | CILP | HLA-A | PCSK5 | SSR1 |  |  |
| IFITM1 |  |  | CLIC4 | HLA-B | PDE3A | SSR3 |  |  |
| IRAK2 |  |  | CMTM3 | HLA-C | PDE7B | STAT1 |  |  |
| JPH2 |  |  | CMTM6 | HLA-F | PDGFC | STAT3 |  |  |
| KIRREL1 |  |  | CNN3 | HLX | PDIA3 | STEAP1 |  |  |
| KLF2 |  |  | COL11A1 | HNMT | PDIA4 | STK38L |  |  |
| KLHL38 |  |  | COL12A1 | HOMER1 | PDIA6 | SUCLG2 |  |  |
| KRT18 |  |  | COL3A1 | HS3ST3A1 | PDLIM1 | SURF4 |  |  |
| KRT8 |  |  | COL4A1 | HSP90B1 | PEA15 | SVIL |  |  |
| LDLRAD2 |  |  | COL4A2 | HSPA5 | PEAK1 | SYDE1 |  |  |
| LGALS3BP |  |  | COL5A2 | HSPB1 | PHLDA3 | SYPL1 |  |  |
| LTBP2 |  |  | COL6A2 | HTRA1 | PHLDB2 | TCF7L2 |  |  |
| LUM |  |  | COL6A3 | HYOU1 | PHTF2 | TFE3 |  |  |
| MAFK |  |  | COPA | ID1 | PIAS3 | TFPI |  |  |
| MICALL2 |  |  | CPED1 | ID3 | PLAGL1 | TGFB1I1 |  |  |
| MLPH |  |  | CPS1 | IFI16 | PLEKHA2 | TGFBR2 |  |  |
| MSLN |  |  | CRABP2 | IFITM2 | PLEKHH2 | THBS1 |  |  |
| MSRB3 |  |  | CREG1 | IGF2R | PLK2 | TIMP1 |  |  |
| MX1 |  |  | CRELD2 | IGFBP5 | PLOD2 | TJP1 |  |  |
| MYH9 |  |  | CRISPLD1 | IL13RA1 | PLOD3 | TM4SF1 |  |  |
| MYO1C |  |  | CRTAP | IL6ST | PLPP1 | TM9SF2 |  |  |
| MYOM1 |  |  | CSRP1 | INSIG1 | PLSCR1 | TMBIM4 |  |  |
| NFIL3 |  |  | CTDSP2 | IQGAP2 | PLSCR4 | TMED9 |  |  |
| NFKB2 |  |  | CTNNA1 | ITGA10 | PLXDC2 | TMEFF2 |  |  |
| NUAK2 |  |  | CTSB | ITGA4 | PON2 | TMEM263 |  |  |
| P4HA2 |  |  | CTSC | ITGAV | POSTN | TMEM50A |  |  |
| P4HB |  |  | CTSO | ITGB1 | PPIB | TMEM87B |  |  |
| PDE6G |  |  | CXCL12 | ITM2B | PPIC | TNC |  |  |
| PDGFA |  |  | CYBRD1 | ITM2C | PPT1 | TNFRSF12A |  |  |
| PGF |  |  | CYFIP1 | ITPR1 | PRCP | TNFRSF1A |  |  |
|  |  |  | CYP26A1 | ITPRIPL2 | PRDM6 | TNMD |  |  |
|  |  |  | CYR61 | JAK1 | PRDX4 | TNS1 |  |  |
|  |  |  | DCAF6 | JAM3 | PRDX6 | TOR1AIP1 |  |  |
|  |  |  | DDOST | KCNK2 | PROM1 | TPBG |  |  |
|  |  |  | DDR2 | KCTD12 | PRRX1 | TPM1 |  |  |
|  |  |  | DESI2 | KDELC2 | PTBP1 | TPM2 |  |  |
|  |  |  | DKK3 | KDELR2 | PTGER4 | TRAM1 |  |  |
|  |  |  | DLC1 | KDELR3 | PTGFRN | TRIL |  |  |
|  |  |  | DLX1 | KDM5B | PTN | TRIM5 |  |  |
|  |  |  | DLX2 | KIAA1462 | PTPN14 | TSC22D2 |  |  |
|  |  |  | DMD | KIF13A | PTPRG | TSC22D3 |  |  |
|  |  |  | DNAJC1 | KIRREL | PTPRK | TUBB6 |  |  |
|  |  |  | DNAJC10 | KLF10 | PTRF | TWSG1 |  |  |
|  |  |  | DNAJC3 | KLF4 | PXDC1 | TXNDC12 |  |  |
|  |  |  | DNM3OS | KLF6 | PXDN | UAP1 |  |  |
|  |  |  | DPY19L1 | L3HYPDH | PYGL | UGDH |  |  |
|  |  |  | DSE | LAMB1 | QKI | VCL |  |  |
|  |  |  | DUSP14 | LAMC1 | QSOX1 | VIM |  |  |
|  |  |  | DUSP5 | LAMP1 | RAB13 | WIPI1 |  |  |
|  |  |  | DUSP6 | LAPTM4A | RAB29 | WLS |  |  |
|  |  |  | EDEM1 | LASP1 | RAB31 | WNT5A |  |  |
|  |  |  | EDNRA | LATS2 | RAP1A | WWTR1 |  |  |
|  |  |  | EGR1 | LEPROT | RAP1B | YAP1 |  |  |
|  |  |  | EGR3 | LGALS1 | RBMS1 | ZNF217 |  |  |
|  |  |  | EHD2 | LHFP | RCN1 |  |  |  |
|  |  |  | ELAVL1 | LHX8 | RECK |  |  |  |
|  |  |  | ELF1 | LIFR | REST |  |  |  |
